# Iron Regulates the Warburg Effect and Ferroptosis in Colorectal Cancer

**DOI:** 10.3389/fonc.2021.614778

**Published:** 2021-05-18

**Authors:** Yin Yuan, Shuo Ni, Aoxiang Zhuge, Bo Li, Lanjuan Li

**Affiliations:** ^1^ State Key Laboratory for Diagnosis and Treatment of Infectious Diseases, The First Affiliated Hospital, School of Medicine, Zhejiang University, Hangzhou, China; ^2^ Department of Orthopedics, Shanghai Pudong Hospital, Fudan University Pudong Medical Center, Shanghai, China; ^3^ Research Units of Infectious Disease and Microecology, Chinese Academy of Medical Sciences, Hangzhou, China

**Keywords:** iron, ROS, NRF2, the Warburg effect, ferroptosis

## Abstract

Iron promotes the proliferation of cancer cells, but it also contributes to cell death. Here we explored whether iron could promote the Warburg effect of colorectal cancer (CRC) cells and suppress sensitivity to ferroptosis by inducing reactive oxygen species (ROS) and regulating nuclear factor erythroid 2-related factor 2 (NRF2). In this study, cell proliferation abilities were measured by CCK-8, EdU incorporation, and colony formation assays. Seahorse XF96 respirometry assays were used to detect the Warburg effect and the level of ROS was assess by DCFH-DA fluorescent probes. Results showed that iron exposure promoted the Warburg effect of CRC cells by inducing ROS and activating NRF2 both *in vivo* and *in vitro*. In addition, iron exposure also induced ferroptosis in CRC cells, but at the same time its inhibitory proteins SLC7A11 and GPX4 were also upregulated, indicating an enhanced resistance to ferroptosis. Our results revealed that iron can effectively promote tumorigenesis. Meanwhile, iron elimination or a low-iron diet might be valid therapeutic approaches for CRC.

## Introduction

Colorectal cancer (CRC) is one of the deadliest cancers worldwide (1). Many unfavorable factors, such as aging, unhealthy dietary habits, genetic predisposition, and inflammation, increase the risk of CRC (2, 3). Iron is an essential substance for the proliferation, differentiation, and even death of cells (4). Iron deficiency could result in diseases, whereas excess iron could also lead to tissue damage through its pro-oxidative effects. Studies examining the correlation between iron intake and CRC have found controversial results. Most studies have reported that iron intakes could elevate the risk of CRC for both men and women (5, 6). However, on the contrary, an epidemiological study on Asian populations has revealed that zinc and heme dietary iron intakes are not associated with the progression of CRC (7). Scientists have speculated that the oxidative stress, lipid peroxidation, and cell cycle alterations induced by iron may contribute to cancer occurrence and progression (8–10), but the mechanism by which iron promotes cancer cell proliferation has not been clearly defined.

Excess intracellular iron can lead to the Fenton reaction, by which large amounts of hydroxyl radicals, a kind of reactive oxygen species (ROS), can be produced (11). Elevated levels of ROS are closely related to carcinogenesis due to damage to chromosomes, lipids, and proteins (12, 13). Antioxidant proteins such as nuclear factor erythroid 2-related factor 2 (NRF2) help to prevent the accumulation of excessive ROS and maintain oxidation-reduction (REDOX) homeostasis. Thus, appropriate levels and functionality of NRF2 are critical in maintaining REDOX homeostasis, especially under conditions of oxidative stress.

The Warburg effect refers to the high level of glycolysis in cancer cells even under aerobic conditions and is recognized as a hallmark of cancer cells (14). Enhanced glycolysis can generate more ATP in a shorter period of time than the TCA cycle, meeting the biosynthetic needs for the uncontrolled proliferation of cancer cells (15, 16). Inhibiting the Warburg effect can result in a reduction in the cell proliferation ability. Therefore, inhibiting the Warburg effect could be a therapeutic strategy for cancer.

Ferroptosis is a unique mode of non-apoptotic cell death first identified by Stockwell et al. in 2012 and characterized by a reduced mitochondrial size and a higher mitochondrial membrane density (17). Ferroptosis is iron-dependent. Studies have shown that iron overload can induce alterations in mitochondrial morphology and acceleration of lipid peroxidation, indicating the occurrence of ferroptosis (17). In contrast, ferroptosis cannot occur after iron elimination by iron-chelating agents. Studies have confirmed that inducing ferroptosis in cancer cells can effectively inhibit tumorigenesis. Thus, ferroptosis can be considered a potential anticancer target.

In this study, we first revealed that iron exposure can facilitate CRC growth by enhancing the Warburg effect and suppressing ferroptosis sensitivity both *in vivo* and *in vitro*. Furthermore, we found that iron exerts these effects by inducing ROS production and activating NRF2 and that these effects are partially rescued after NRF2 knockdown. Our study is the first to clarify the relationship between iron, the Warburg effect, and ROS in CRC, providing a novel pathological perspective for CRC.

## Materials and Methods

### Cell Lines and Reagents

The human CRC cell lines HCT-116 and HT-29 were purchased from the American Type Culture Collection (ATCC; Manassas, VA, USA) and cultured in RPMI 1640 medium containing 10% fetal bovine serum (FBS; Gibco, Gaithersburg, MD, USA). Ferric ammonium citrate (FAC) was purchased from Aladdin (Shanghai, China), and deferoxamine (DFO) was purchased from MedChem Express (MCE; Monmouth Junction, NJ, USA).

### Cell Viability

Cell viability was measured with an enhanced CCK-8 assay (Beyotime, Shanghai, China). Firstly, human CRC cells HCT-116 and HT-29 were incubated in 96-well plates at a density of 5,000 cells per well overnight. Next, for dose dependence assays, HCT-116 and HT-29 cells were stimulated with 0–1,000 µM FAC for 12 h. For time dependence assays, cells were treated with 100 µM FAC or 100 µM DFO for 0–48 h. Then CCK-8 solutions were added to each sample and all samples were incubated for 1.5 h. The absorbance was measured at 450 nm. For each condition, three independent biological duplicates were performed.

### EdU Incorporation Assay

An EdU incorporation kit (Beyotime) was used to measure the cell proliferation ability. Cells pretreated with 100 µM FAC or 100 µM DFO for 12 h were labeled with EdU and stained with Hoechst 33342. Images were acquired using an Olympus FSX100 microscope (Olympus, Tokyo, Japan).

### Colony Formation

Cells were plated in six-well plates (400 cells per well) in a sufficient volume of RPMI 1640 medium containing FAC or DFO. All plates were incubated for 14 days, and colonies were then fixed with methane and stained with Giemsa solution (Solarbio, Beijing, China).

### Glucose Uptake, Lactate Production, and ATP Content Assays

Glucose uptake, L-lactate production, and ATP content assay kits were purchased from Abcam (Cambridge, MA, USA). All experiments were performed in accordance with the kit instructions. Briefly, CRC cells were planted into 96-well plates at a density of 3,000 cells per well and stimulated with FAC (100 µM) or DFO (100 µM) for 12 h. For glucose uptake assay, after incubated with 2-DG for 20 min, cells were washed with PBS, counted, and then lysed with extraction buffers. Then supernatants were mixed with reaction reagents. OD values were measured at 412 nm. For lactate production and ATP assay, we collected the supernatant medium of each well and mixed it with kit-provided reaction reagents. Results were measured at the absorption maximum wavelength specified by the kit (450 nm for lactate production and 570 nm for ATP assay). All results were quantified against corresponding standard curves. Three independent duplicates were performed for each condition. Data were normalized to total live cell numbers and presented as fold change to control group.

### Extracellular Acidification Rate (ECAR) and Oxygen Consumption Rate (OCR) Assays

The ECAR and OCR values were measured by using an XF96 extracellular flux analyzer (Seahorse Bioscience) in accordance to the manufacturer’s instructions. Briefly, HCT-116 and HT-29 cells were plated in XF96 seahorse plates at the density of 2 × 10^4^ cells per well and treated with 100 µM FAC or DFO 12 h before assessment. Then, for OCR measurement, 1 µM oligomycin, 0.5 µM FCCP, and 1 µM rotenone/antimycin A (AA) were sequentially added into plates. For ECAR measurement, 10 mM glucose, 1 µM oligomycin, and 100 mM 2-DG were automatically injected into plates at specific time points. Data were analyzed by Wave software. Results were normalized to cell number and three independent duplicates were performed for each condition.

### Lipid Peroxidation Assay

The lipid peroxidation was detected by C11-BODIPY (Invitrogen). Briefly, cells were harvested after different treatments and incubated with 2 uM C11-BODIPY for 20 min at 37°C. Images were acquired with an LSM T-PMT confocal microscope (Zeiss, Jena, Germany).

### ROS Production

Intracellular ROS production was detected with an ROS assay kit (Beyotime). In accordance with the kit protocol, after pre-treated with 100 µM FAC for 12 h, cells were collected and then incubated with 10 µM DCFH-DA fluorescent probe for 30 min. Fluorescence intensity values were measured by flow cytometry (BD Biosciences Franklin Lakes, NJ, USA).

### Mitochondrial ROS Assessment

A Mito-Sox™ red mitochondrial superoxide indicator (Invitrogen, CA, USA) was used to determine ROS in mitochondria. Briefly, cells were seeded in 24-well plate overnight and treated with 100 µM FAC for 12 h. Then all cells were incubated with 5 µM Mito-Sox for 15 min. Images were acquired with an LSM T-PMT confocal microscope (Zeiss).

### Iron Assessment

Iron was detected by a commercial iron assay kit (Abcam ab83366, USA) according to the manufacturer’s protocol. Data were normalized by protein content. In brief, cells were washed three times by HBSS and incubated with calcein (Beyotime, China) at the ratio of 1:1,000 for 30 min. Then all the cells were washed by HBSS twice before assessment. The differences of the iron concentrations from treated or non-treated cells were measured by fluorescence microplates.

### Intracellular Glutathione (GSH) Quantification Assays

Intracellular GSH level was assessed by an intracellular GSH detection assay kit (Abcam). Briefly, transfected cells were seeded in a 96-well plate at the density of 10^4^ per well overnight and treated with 100 µM FAC for 12 h before assessment. The intracellular GSH level was measured in accordance with the manufacturer’s instruction. Results were measured by flow cytometry (BD Biosciences).

### Transfection

The shRNA construct was designed and synthesized by Ribobio Co., Ltd (Guangzhou, China), and its sequence is listed in **Table S1**. For NRF2 knockdown, shRNA was used to generate RNAi stable cell lines. Briefly, HEK293T cells were transfected with lenti-shRNA. After 48 h of incubation, culture medium containing lentivirus was used to infect HCT-116 and HT-29 cells. Lipofectamine 3000 was used in the transfection procedure. Procedure of the generation of Nrf2 over-expression cell lines was the same as shRNA, and the NRF2 overexpression plasmid was constructed by Ribobio Co., Ltd too.

### Western Blotting

Proteins were collected from cells and mice tissues by using RIPA lysis buffer. After separation by SDS-PAGE, proteins were transferred to PVDF membranes and detected with specific antibodies. Antibodies specific for NRF2 (#12721), HO-1 (#43966), LDHA (#2012), PGK1 (#68540), HK2 (#2867), and SLC7A11 (#12691) were purchased from CST (Beverly, MA, USA); an antibody specific for GPX4 (ab125066) was purchased from Abcam; and antibodies specific for α-tubulin (BM1452) and β-actin (BM0627) were purchased from Boster Biological Technology (Wuhan, China).

### Immunofluorescence

According to protocols, pretreated cells were fixed with methanal and blocked with serum. Then, the cells were incubated with specific primary antibodies overnight and with secondary antibodies for 1 h. Images were acquired with an LSM T-PMT confocal microscope (Zeiss).

### RNA Extraction and qRT-PCR

RNeasy Mini Kits (Qiagen, Valencia, USA) were used to extract RNA from pretreated cells. Then, the RNA was analyzed in a VII A7 real-time PCR system (Applied Biosystems, Foster, CA, USA). The sequences of the primers are listed in **Table S1**.

### Chromatin Immunoprecipitation (ChIP) Assay

ChIP assay was performed with a Chromatin immunoprecipitation (ChIP) assay kit (Sigma Aldrich, St. Louis, MO, USA). In brief, chromatin was cross-linked with methanol and then lysed into small fragments. Then samples were immunoprecipitated with NRF2 antibody. IgG antibody was used as a negative control. Primers for ChIP assays were designed by using Primer 3 program. DNA fragments were assessed by qRT-PCR.

### Transmission Electron Microscopy

Cell samples were first fixed with 2.5% glutaraldehyde and then double-fixed with 1% osmium tetroxide. After gradient dehydration and embedding in epoxy resin, samples were cut into small pieces and imaged with a transmission electron microscope (TEM; Hitachi, Tokyo, Japan).

### Xenograft Mouse Models

Six-week-old BALB/c athymic nude mice were injected subcutaneously with HT-29 cells. After 7 days, all mice were randomly divided into two groups. The high-iron group was fed a high-iron diet (HID; 350 ppm iron), and the control group was fed a standard diet (with sufficient iron, approximately 35 ppm) for 7 days. All diets were purchased from Test Diet (Richmond, IN). Tumor volumes were measured every 2 days and were calculated based on the formula “Tumor volume = length * width^2^/2” (18). All animal experiments were conducted in accordance with the guidelines of the Animal Care Committee of Zhejiang University School of Medicine.

### Metabolome Analysis

According to protocols, metabolites were extracted with cold methanol and were then analyzed by LC-MS/MS. Chromatographic peak areas, representing the abundances of the metabolites, were calculated.

### Statistical Analysis

SPSS software (Chicago, IL, USA) was used to analyze data, and GraphPad Prism 7.0 (San Diego, CA, USA) was used to generate figures. ANOVA and Student’s t-test were used to evaluate differences between groups. The results are presented as the means ± SEMs, and p values of < 0.05 were considered significant.

## Results

### HID Feeding Increased the Tumor Burden and Activated Glycolysis *In Vivo*

**Figure 1:**
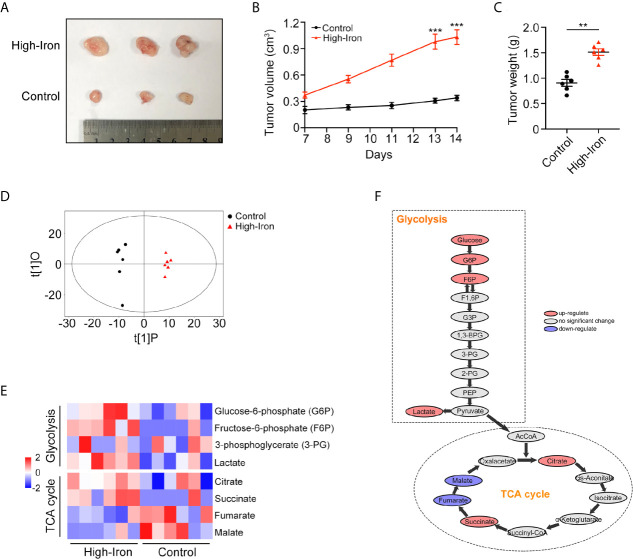
High-iron diet (HID) promoted tumorigenesis and enhanced glycolysis. **(A–C)** Photos, volumes, and weights of tumors. Mice in High-Iron group were fed with HID for 7 days. **(D)** OPLS-DA scores of the control group and the HID group. **(E, F)** Metabolite shifts of glycolysis and TCA cycle in CRC. (**p < 0.01; ***p < 0.001).

Accumulating epidemiological evidence suggests that iron can promote tumorigenesis (11). In this study, we used an HT-29 xenograft mouse model to explore whether HID feeding can promote the progression of CRC. Both the weight and volume of tumors were higher in the HID group than in the control group (p < 0.01) (**Figures 1A–C**), indicating that high iron level can increase the tumor burden in mouse models. For further exploration, we conducted a metabolome analysis and found a significant separation between the control group and the HID group based on the orthogonal projections to latent structures discriminant analysis (OPLS-DA) scores (**Figure 1D**). The abundances of the glycolytic metabolites G6P, F6P, and lactate were higher in the HID group than in the control group, suggesting that HID can intensify glycolytic pathway activity in CRC. In addition, the abundances of the TCA cycle metabolites fumarate and malate were lower in the HID group, indicating that the rate of oxidative phosphorylation was decreased (**Figures 1E, F**). High levels of glycolysis and low levels of TCA cycle-mediated oxidative phosphorylation are typical characteristics of the Warburg effect (19). Thus, we speculated that HID feeding can promote the Warburg effect and thus exacerbate tumorigenesis.

### Iron Promoted CRC Cell Proliferation *In Vitro*

**Figure 2:**
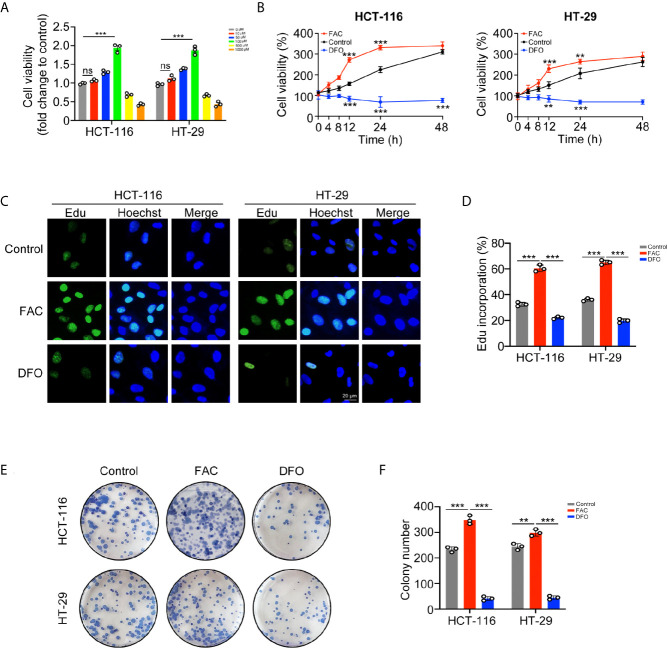
Iron promoted CRC cells proliferation and colony formation *in vitro*. **(A)** Viabilities of cells treated with different concentrations of FAC (0–1,000 µM) for 12 h. **(B)** Viabilities of cells treated with FAC (100 µM) or DFO (100 µM) for 0–48 h **(C, D)** Immunofluorescence images and percentages of Edu incorporation. **(E, F)** Colony images and numbers of HCT-116 and HT-29 cells. Cells were incubated in medium containing 100 µM FAC or 100 µM DFO for 14 days. (**p < 0.01; ***p < 0.001; ns, not significant).

*In vitro* experiments, we stimulated HCT-116 and HT-29 CRC cells with FAC, the iron chelator DFO, and PBS to explore whether iron exposure can promote the proliferation of CRC cells. Firstly, we compared the viabilities of CRC cell following treatment with different concentrations (0–1,000 µM) of FAC. Cell viabilities gradually increased within the concentration of 0–100 µM but decreased significantly upon the concentration of 500 and 1,000 µM (**Figure 2A**). Given that high level of FAC could induce widespread cell death (20), we considered 100 µM FAC as an optimal concentration for further study. Next, we compared the viabilities of cells treated with 100 µM FAC or DFO in different time periods (0–48 h). Interestingly, viabilities of FAC-treated cells increased rapidly from 0 to 12 h, but the increasing speed slowed down when cells were treated with FAC for more than 24 h. Viabilities of those DFO-treated cells decreased persistently from 0 to 48 h (p < 0.001) (**Figure 2B**). Furthermore, we performed a series of experiments to explore the influence of DFO. After adding 100 uM FAC or DFO to cell medium, we measured the iron concentrations both in medium and intracellular. Besides, given that 100 uM DFO chelated Fe3+ at the ratio of 1:1, we also added 200 uM FAC to the DFO group in order to explore whether the inhibitory effect of DFO could be rescued by double dose of iron. As **Figure S1** showed, no natural presence of iron was detected in RPMI 1640 medium. The 100 uM FAC significantly increased intracellular iron level while 100 uM DFO reduced it (p < 0.001). Double dose of FAC could rescue intracellular iron concentrations and cell viabilities of HCT-116 and HT-29 (p < 0.001) (**Figure S1**). These results indicate that DFO exerts its inhibitory effect by decreasing intracellular iron concentration. The results of the EdU cell proliferation assay also showed that FAC increased the percentage of EdU-positive cells, while the percentage decreased after complete chelation of iron by DFO (p < 0.001) (**Figures 2C, D**). In addition, FAC enhanced and DFO reduced the colony-forming ability of both cell lines (**Figures 2E, F**). Collectively, these results showed that iron exposure can enhance the proliferative ability of CRC cells *in vitro*.

### Iron Promoted the Warburg Effect in CRC Cells *In Vitro*

**Figure 3:**
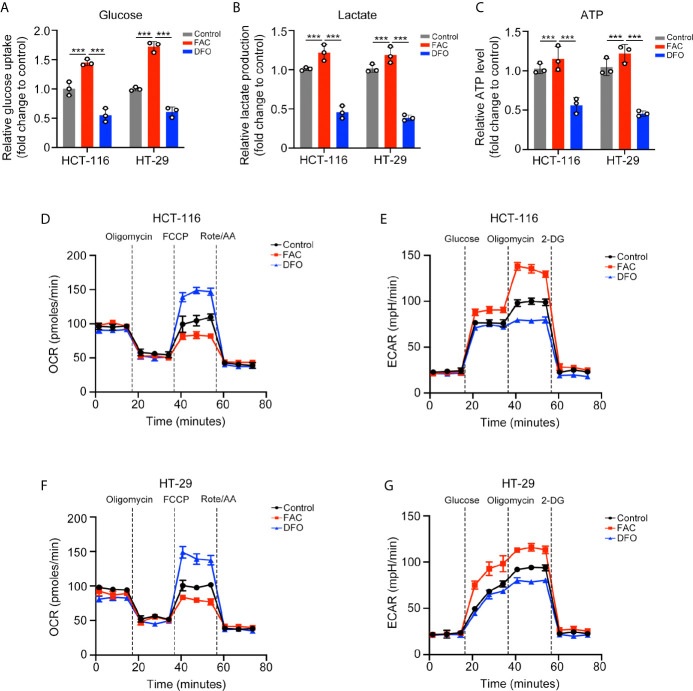
Iron promoted the Warburg effect in CRC cells. **(A–C)** Glucose uptake, lactate production, and ATP level of cells treated with FAC (100 µM) or DFO (100 µM) for 12 h. **(D–G)** Oxygen consumption rate (OCR) and extracellular acidification rate (ECAR) values of both cell lines pre-treated with 100 µM FAC or DFO for 12 h. (***p < 0.001).

We further explored the potential mechanism by which iron promotes cell proliferation. Cancer cells can acquire considerable amounts of energy through glycolysis even under aerobic conditions to meet the requirement of uncontrolled growth and this phenomenon is called the Warburg effect (15). We evaluated whether iron can regulate the Warburg effect in CRC cells. FAC increased while DFO reduced the rates of glucose uptake and lactate production and the content of ATP in both cell lines (p < 0.001) (**Figures 3A–C**). Furthermore, we measured the ECAR and OCR by using an extracellular flux analyzer. In both cell lines, FAC significantly increased the ECAR and reduced the OCR, reflecting enhancement of glycolysis and downregulation of mitochondrial respiration, respectively. The DFO group exhibited the opposite results (**Figures 3D–G**). Taking these results together, we concluded that iron can promote the Warburg effect, increase energy production and, thus, accelerate cell proliferation.

### Iron Exposure Induced Mitochondrial ROS Production and Activated the NRF2 Pathway

**Figure 4:**
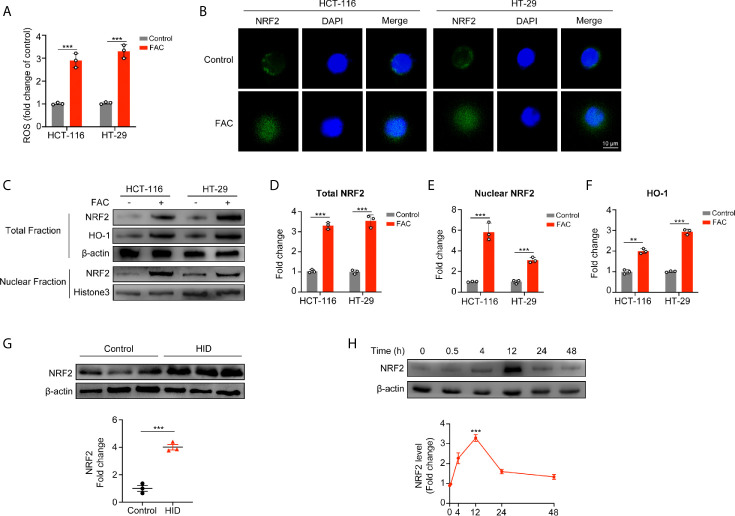
Acute iron exposure produced ROS and activated NRF2. **(A)** Reactive oxygen species (ROS) production in cells pre-treated with 100 µM FAC for 12 h. **(B)** Immunostaining of NRF2 (green) and DAPI (blue). HCT-116 and HT-29 cells were treated with or without 100 µM FAC for 12 h. Images were acquired by a confocal microscope. **(C–F)** Western blot bands and quantitative analysis of total and nuclear NRF2 as well as downstream protein HO-1 in HCT-116 and HT-29 cells. Cells were treated with or without 100 µM FAC for 12 h before protein extraction. **(G)** Western blot analysis of NRF2 in mice tissues. **(H)** NRF2 expression and quantitative analysis of HCT-116 and HT-29 cells treated with FAC in different time periods. (**p < 0.01; ***p < 0.001).

Next, we evaluated intracellular ROS production and found that FAC increased the intracellular ROS level (p < 0.001) (**Figure 4A**), and this iron-induced ROS was produced mainly by mitochondria (**Figure S2A, B**). Given that NRF2 is a major antioxidant transcription factor, we measured the protein level of NRF2 both in cellular cytoplasm and in nucleus. Surprisingly, FAC treatment increased the protein level of total NRF2 (p < 0.001). Moreover, comparing to the control group, nuclear NRF2 protein level was also significantly increased with FAC treatment, and the fluorescence intensity of NRF2 in nucleus was significantly increased (p < 0.001) (**Figures 4B–E**). Given that NRF2 must exerts its function in nucleus, we concluded that FAC could promote the expression of NRF2, significantly increase the level of nuclear NRF2, and thus activate NRF2. Besides, HO-1, a downstream protein of NRF2, was also increased with FAC treatment, indicating that the NRF2 was activated (p < 0.001) (**Figure 4F**). The level of NRF2 was also increased in tissues from mice being fed with HID (**Figure 4G**). However, we found that being stimulated with FAC for different time periods differed the expression of NRF2. The level of NRF2 did not change significantly at 30 min, but it increased lightly at 4 h and reached the maximum at 12 h. However, NRF2 level decreased significantly when cells were stimulated for more than 24 h, and NRF2 was almost depleted when cells were subjected to 48 h exposure (**Figure 4H**). We speculated that this phenomenon might have occurred because the FAC-induced ROS had not been accumulated in cells at 30 min and 4 h but accumulated excessively at 48 h. More details were discussed in the discussion section.

### Knockdown of NRF2 Conferred Resistance Against Iron-Induced Cell Proliferation

**Figure 5:**
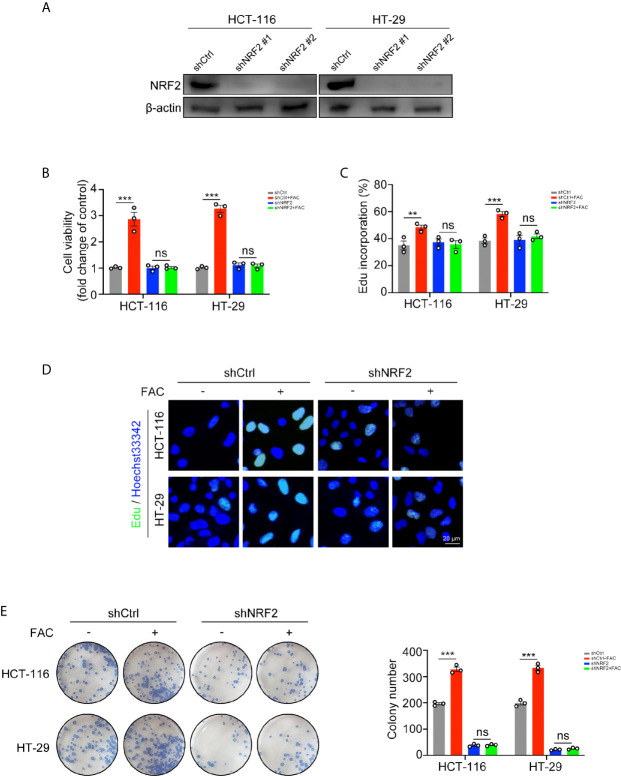
Iron-induced cell proliferation was rescued by NRF2 knockdown. **(A)** Western blots of HCT-116 and HT-29 cells transfected with control shRNA (shCtrl) and NRF2 shRNA (shNRF2). shNRF2#1 was used in following studies for its better knockdown effect. **(B)** Cell viabilities were assessed by CCK-8 kits. Cells were transfected and then treated with FAC (100 µM) or DFO (100 µM) for 12 h. **(C, D)** Edu assay of transfected HCT-116 and HT-29 cells. Cells were pre-treated with or without 100 µM FAC for 12 h before Edu assay. **(E)** Colony formation assay of transfected HCT-116 and HT-29 cells. Cells were incubated in medium containing 100 µM FAC or equivalent PBS for 14 days. (**p < 0.01; ***p < 0.001; ns, not significant).

Since iron can activate NRF2, we used shRNA to knock down NRF2 in rescue experiments (**Figure 5A**). FAC increased cell viabilities in the control (shCtrl) group while this increasement was partially reversed when NRF2 was knocked down (**Figure 5B**). Similar results were found in the EdU incorporation assays and colony formation assays. The percentage of EdU-positive cells and number of colonies in the control group increased after FAC treatment but did not change significantly after NRF2 knockdown (**Figures 5C–E**). These results suggested that iron exposure promote the growth of CRC cells by activating NRF2.

### NRF2 Activation Promoted the Warburg Effect by Upregulating Its Key Enzymes

**Figure 6:**
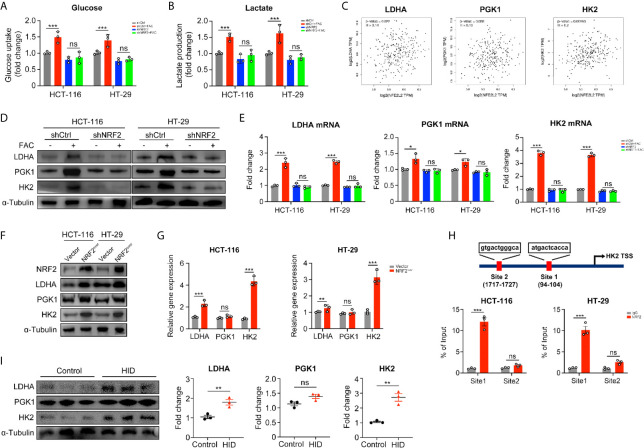
NRF2 knockdown inhibited the Warburg effect by downregulating its key enzymes. **(A, B)** Relative glucose uptake and lactate production level of transfected cells. Cells were treated with or without 100 µM FAC for 12 h. **(C)** Correlations between NRF2 (NFE2L2) and the Warburg effect key enzymes. Data were from TCGA database and analyzed by the GEPIA webtool. **(D)** Western blots of the Warburg enzymes in transfected HCT-116 and HT-29 cells. Cells were treated with or without 100 µM FAC for 12 h before Western blot analysis. **(E)** The mRNA expression levels of the Warburg enzymes in transfected cells treated with or without 100 µM FAC for 12 h. **(F, G)** Western blots and qRT-PCR analysis of HCT-116 and HT-29 cells transfected with empty vector (Vector) and NRF2 vector (NRF2^over^). **(H)** Chromatin immunoprecipitation (ChIP) results of the NRF2 binding sites on HK2 promoter in both HCT-116 and HT-29 cells. **(I)** Western blotting bands and quantitative analysis of Warburg enzymes LDHA, PGK1, and HK2 in mice tissues. (*p < 0.05; **p < 0.01; ***p < 0.001; ns, not significant).

In the above studies, we found that iron can promote the Warburg effect in CRC cells. However, the rates of glucose uptake and lactate production in NRF2 knocked down cells remained unchanged with FAC treatment (p > 0.05) (**Figures 6A, B**). According to the GEPIA2 web tool (http://gepia.cancer-pku.cn/) based on the TCGA database, the NRF2 (NFE2L2) gene and genes of key enzymes mediating the Warburg effect (LDHA, PGK1, and HK2) are positively correlated in colon adenocarcinoma (COAD) patients (**Figure 6C**). Therefore, we hypothesized that iron exposure can increase the expression of Warburg effect enzymes by activating NRF2. However, the Pearson correlation analysis based on TCGA database and GEPIA2 web tool only revealed a very weak correlation between NRF2 and Warburg enzymes (LDHA, PGK1, and HK2). Therefore, in order to verify the correlation, we tested the mRNA and protein levels of LDHA, PGK1, and HK2 in NRF2 knockout cells as well as in NRF2 overexpressed cells. The western blot and qRT-PCR results revealed that FAC treatment increased both the gene expression and protein levels of LDHA and HK2 in shCtrl cells (p < 0.001), but this increasement was abolished in shNRF2 cells (p > 0.05) (**Figures 6D, E**). However, the mRNA expression of PGK1 remained almost unchanged, indicating a very low correlation between PGK1 and NRF2. For further verification, we also detected the levels of these three enzymes in NRF2 overexpressed cells by Western Blot analysis and qRT-PCR. Both LDHA and HK2 proteins increased significantly in NRF2 overexpressed cells, whereas PGK1 remained almost unchanged (**Figures 6F, G**). Given that NRF2 is a major transcriptional factor, we further hypothesized that NRF2 might functions as a transcriptional regulator to Warburg enzymes. Among these three kinds of enzymes, HK2 exhibited the most significant change while PGK1 remained almost unchanged. Therefore, we chose HK2 for ChIP assay in order to explore whether NRF2 could transcriptionally regulate HK2. According to bioinformatics analysis of UCSC gene database (http://www.genome.ucsc.edu/) and JASPAR database (http://jaspar.genereg.net/), there are two potential binding sites for NRF2 on the upstream promoter region of HK2. ChIP assay results showed that NRF2 could bind at site 1 on the HK2 promoter region (**Figure 6H**). These results suggested a possible mechanism by which NRF2 regulates HK2. Besides, we also verified above results in *in vivo* experiment. Western blot results showed that HID feeding *in vivo* increased the protein levels of LDHA and HK2 (p < 0.01), but had little impact on PGK1 (p > 0.05) (**Figure 6I**).

### NRF2 Activation Counteracted the Iron-Induced Lipid Peroxidation and Protected CRC Cells From Ferroptosis

**Figure 7:**
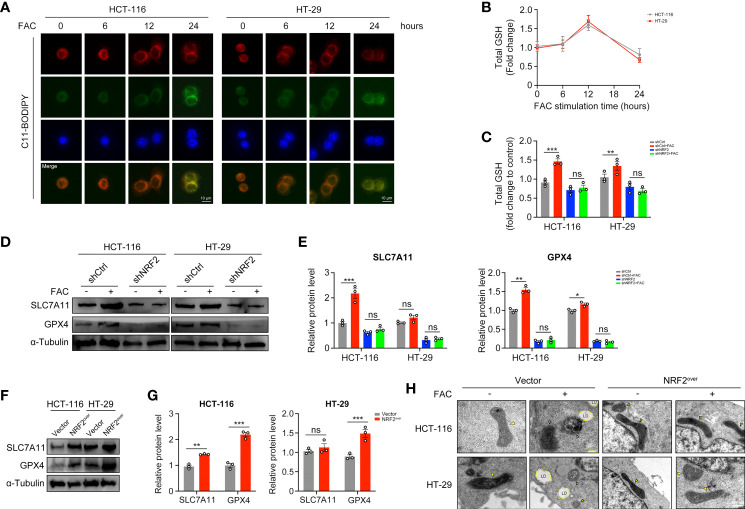
NRF2 activation counteracted the iron-induced lipid peroxidation and protected CRC cells from ferroptosis. **(A)** C11-BODIPY was used to measure lipid peroxidation in HCT-116 and HT-29 cells at different time periods. **(B)** The intracellular GSH level in HCT-116 and HT-29 cells treated with 100 µM FAC for different time. **(C)** The intracellular GSH level in transfected cells upon treatment with 100 µM FAC for 12 h. **(D–G)** Western blots and quantitative analysis of SLC7A11 and GPX4 in NRF2-knockdown cells and NRF2-overexpressed cells. Cells were treated with or without 100 µM FAC for 12 h before Western blot analysis. **(H)** TEM images of control and NRF2 overexpressed cells treated with 100 µM FAC for 12 h. Yellow arrows refer to swelled mitochondrial and LD refers to lipid droplet. (*p < 0.05; **p < 0.01; ***p < 0.001; ns, not significant).

Ferroptosis is a kind of iron-dependent, non-apoptotic cell death. According to studies reported by Dixon et al., iron exposure could induce ferroptosis in cells (17). However, we found that 12-h-treatment of FAC failed to induce significant lipid peroxidation (a hallmark change of ferroptosis) in both HCT-116 and HT-29 cells, whereas 24-h-treatment succeeded (**Figure 7A**). GSH is the major kind of substance that reduces lipid peroxidation, thus we detected the levels of GSH at different time periods. Interestingly, intracellular GSH level increased significantly in cells treated with FAC for 12 h, but this increasement was abolished in shNRF2 cells (**Figures 7B, C**). Levels of intracellular GSH are correlated with cystine-glutamate transporter Xc- (SLC7A11) and GPX4 (21). We tested these two proteins both with Western Blot analysis. Results showed that in HCT-116 cells, FAC increased the levels of SLC7A11 and GPX4 by activating NRF2 and that knockdown of NRF2 partially reversed this increasement (p < 0.01). However, the above-mentioned alteration was non-significant in HT-29 cells (p > 0.05) (**Figures 7D, E**). In addition, we also assessed protein levels of SLC7A11 and GPX4 in NRF2 overexpressed cells and similar results were observed (**Figures 7F, G**). Moreover, under a TEM, no changes in mitochondrial morphology (yellow arrow) and no formation of lipid droplets (LDs) were observed in NRF2-overexpressed cells, revealing that these cells did not undergo ferroptosis when they were treated with FAC (**Figure 7H**). These results in together implied that in those cells stimulated with FAC for 12 h, activated NRF2 could counteract the iron-induced lipid peroxidation by increasing GSH, thus protected cells from ferroptosis.

## Discussion

Iron is an essential element for biological processes, such as DNA replication, cell cycle progression, and energy production and consumption. Iron overload is related to tumorigenesis (22). However, the mechanism by which iron promotes cancer has not yet been fully revealed. In this study, we found that mice fed with a HID tended to have higher tumor burdens than mice fed a standard diet. Furthermore, we found that the glycolysis metabolic pathway was significantly activated in the HID group, suggesting that iron may facilitate tumor growth by influencing tumor metabolism. Thus, a further series of studies were carried out.

The Warburg effect is a metabolic abnormality in which cancer cells can generate large amounts of energy through glycolysis even under aerobic conditions (23). ROS are a group of oxygenic molecules produced by cells under oxidative stress. Studies have revealed that ROS can affect cell metabolism and mitochondrial function, thus influencing the Warburg effect either directly or indirectly (24). In this study, we found that FAC significantly increased the proliferative ability of CRC cells. In addition, the FAC group exhibited higher ECAR values and lower OCR values than the control group, indicating enhancement of the Warburg effect. Furthermore, we also found that FAC induced the production of mitochondrial ROS in CRC cells and that this iron-induced ROS activated the expression of NRF2 in nucleus, a key regulator of ROS. These results indicated that iron may promote the Warburg effect by inducing ROS production and activating NRF2. Thus, we carried out a rescue experiment. In NRF2 knockdown cells, iron no longer promoted cell proliferation, and the Warburg effect was suppressed compared with that in wild-type cells. Next, we searched the TCGA database and found that the NRF2 (NFE2L2) gene was positively correlated with the Warburg enzymes LDHA, PGK1, and HK2 in colon adenocarcinoma. However, the Pearson correlation analysis based on TCGA database and GEPIA2 web tool only revealed a very weak correlation between NRF2 and Warburg enzymes (LDHA, PGK1, and HK2). Therefore, in order to verify the correlation, we tested the mRNA and protein levels of LDHA, PGK1, and HK2 in NRF2 knockout cells as well as in NRF2 overexpressed cells. Based on a series of experiments, we found that FAC significantly increased the expression of HK2 by activating NRF2. Moreover, we also found a binding site for NRF2 on the promoter region of HK2 by ChIP assays, which further identified how NRF2 functioned as a transcriptional regulator of HK2. However, we performed ChIP assays only for HK2 in this study and the ChIP results of HK2 could not represent other kinds of Warburg enzymes. To determine whether NRF2 functions as a universal transcriptional regulator to Warburg effect enzymes, further studies are needed.

Interestingly, different time periods of FAC treatment differs the level of NRF2. We found that 30 min and 4 h of FAC treatment did not increase NRF2 level significantly but 12 h did. On the contrary, 48 h of iron exposure resulted in a NRF2 deficiency. Viabilities of FAC-treated cells increased rapidly from 0 to 12 h, but the increasing speed slowed down when cells were treated for more than 24 h. However, viabilities of those DFO-treated cells decreased persistently from 0 to 48 h. We attributed these conflicting results to the Fenton reaction. Free iron can easily accept and donate electrons, and catalyze the Fenton reaction to generate reactive oxygen species (ROS) (25). Iron induced compensable ROS through Fenton reaction and thus activated NRF2 and promoted Warburg effect as well as proliferation of CRC cells when cells were treated with FAC for less than 12 h. Compensable oxidative stress induced by iron exposure could result in a compensatory reaction in cells, activating the protective antioxidant protein NRF2. This activated NRF2 could then protect cells from damage caused by various electrophilic and oxygenic substances. However, excessive ROS were accumulated when cells were stimulated with FAC for more than 24 h. Decompensation of REDOX occurred in cells exposed to excessive ROS and this decompensation resulted in depletion of NRF2, thus the cell viabilities remained almost unchanged. NRF2 participates not only in regulating intracellular REDOX homeostasis but also in many biological processes. More explorations are still needed.

Ferroptosis is a kind of cell death characterized by lipid peroxidation and iron dependence. In cells exposed to high levels of iron, excess intracellular iron consumes a large amount of glutathione (GSH, a major intracellular reducing agent), causing lipid peroxidation and thus resulting in ferroptosis (26, 27). The level of intracellular GSH is associated with cystine-glutamate transporter Xc^−^ (SLC7A11), and inhibition of SLC7A11 usually leads to the depletion of GSH, thus induces ferroptosis. Moreover, GSH also functions as a co-factor of GPX4, which also plays an important role in determining the level of lipid oxidation in cytoplasm (21). Interestingly, in this study we found that FAC increased the levels of SLC7A11 and GPX4 by activating NRF2 and that knockdown of NRF2 partially reversed this increasement in HCT-116 cells, but this kind of increasement was non-significant in HT-29 cells. We attributed the difference to cancer heterogeneity that HT-29 exerted less sensitivity to iron exposure and we will work on this problem in our future studies.

**Figure 8:**
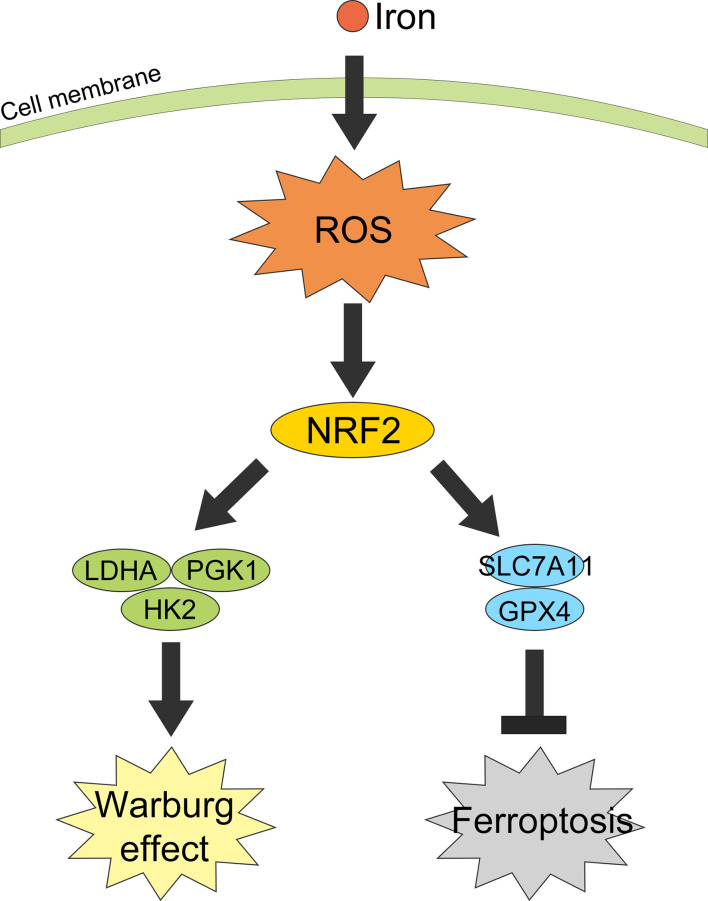
Graphical abstract of iron promotes the Warburg effect and reduces ferroptosis sensitivity by inducing ROS and activating NRF2.

Collectively, in this study we revealed iron exposure could activate ROS and NRF2, resulting in an increasement of Warburg enzymes (LDHA, PGK1, and HK2) and GSH-related proteins SLC7A11 and GPX4, thus enhancing the Warburg effect, counteracting the iron-induced lipid peroxidation and protecting CRC cells from ferroptosis (**Figure 8**).

## Conclusion

Iron-induced ROS promotes CRC cells proliferation by enhancing their Warburg effect both *in vivo* and *in vitro*. Moreover, this iron-induced ROS activated the expression of NRF2 in nucleus and thus upregulated the expression of Warburg enzymes. Besides, short-time exposure to iron increased GSH-related proteins SLC7A11 and GPX4, thus counteracting the iron-induced lipid peroxidation and protected CRC cells from ferroptosis. These results revealed that high iron was a risk factor of CRC tumorigenesis.

## Data Availability Statement

The original contributions presented in the study are included in the article/**Supplementary Material**. Further inquiries can be directed to the corresponding author.

## Ethics Statement

All animal experiments were conducted in accordance with the guidelines of the Animal Care Committee of Zhejiang University School of Medicine.

## Author Contributions

YY and SN designed the study, performed the experiments, and drafted the manuscript. AZ and BL analyzed the data. LL revised the paper. All authors contributed to the article and approved the submitted version.

## Funding

This work was supported by the National Natural Science Foundation of China (81790631 and 81703430).

## Conflict of Interest

The authors declare that the research was conducted in the absence of any commercial or financial relationships that could be construed as a potential conflict of interest.

## Supplementary Material

Supplementary Figure 1 DFO exerts its inhibitory effect by decreasing intracellular iron concentration. **(A, B)** Medium and intracellular iron concentrations in different groups. Cell medium were supplemented with 100 uM FAC (FAC group), 100 uM DFO (DFO group), 100 uM DFO along with 200 uM FAC (DFO+2xFAC group) and equivalent PBS (PBS group). **(C)** Cell viabilities of different groups assessed by CCK-8 assays. (***p < 0.001).

Supplementary Figure 2 Iron-induced ROS mainly accumulated in mitochondria. **(A, B)** Mitochondria ROS production detected by MitoSox (red) and Hoechst 33342 (blue) and its quantitative analysis. (***p < 0.001).

Supplementary Table 1 Primers for qRT-PCR and sequences for cell transfection.
