# Supplementary figures and images for: Iron Regulates the Warburg Effect and Ferroptosis in Colorectal Cancer
Source: Front Oncol. 2021 May 18;11:614778. doi: 10.3389/fonc.2021.614778 (PMC8169994; doi:10.3389/fonc.2021.614778)

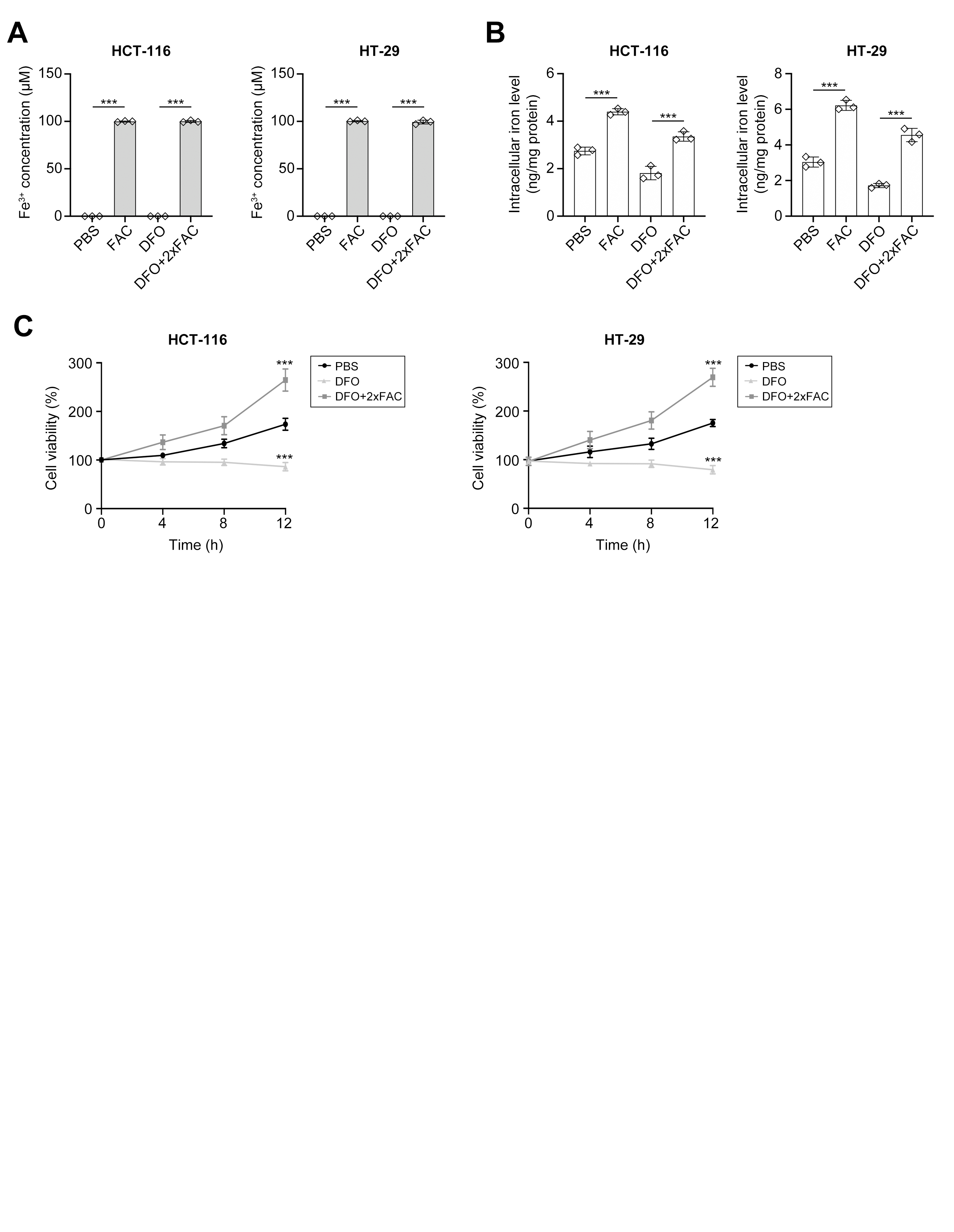

Supplement: Supplementary Figure 1 — DFO exerts its inhibitory effect by decreasing intracellular iron concentration. (A, B) Medium and intracellular iron concentrations in different groups. Cell medium were supplemented with 100 uM FAC (FAC group), 100 uM DFO (DFO group), 100 uM DFO along with 200 uM FAC (DFO+2xFAC group) and equivalent PBS (PBS group). (C) Cell viabilities of different groups assessed by CCK-8 assays. (***p < 0.001). [file Image_1.tif]

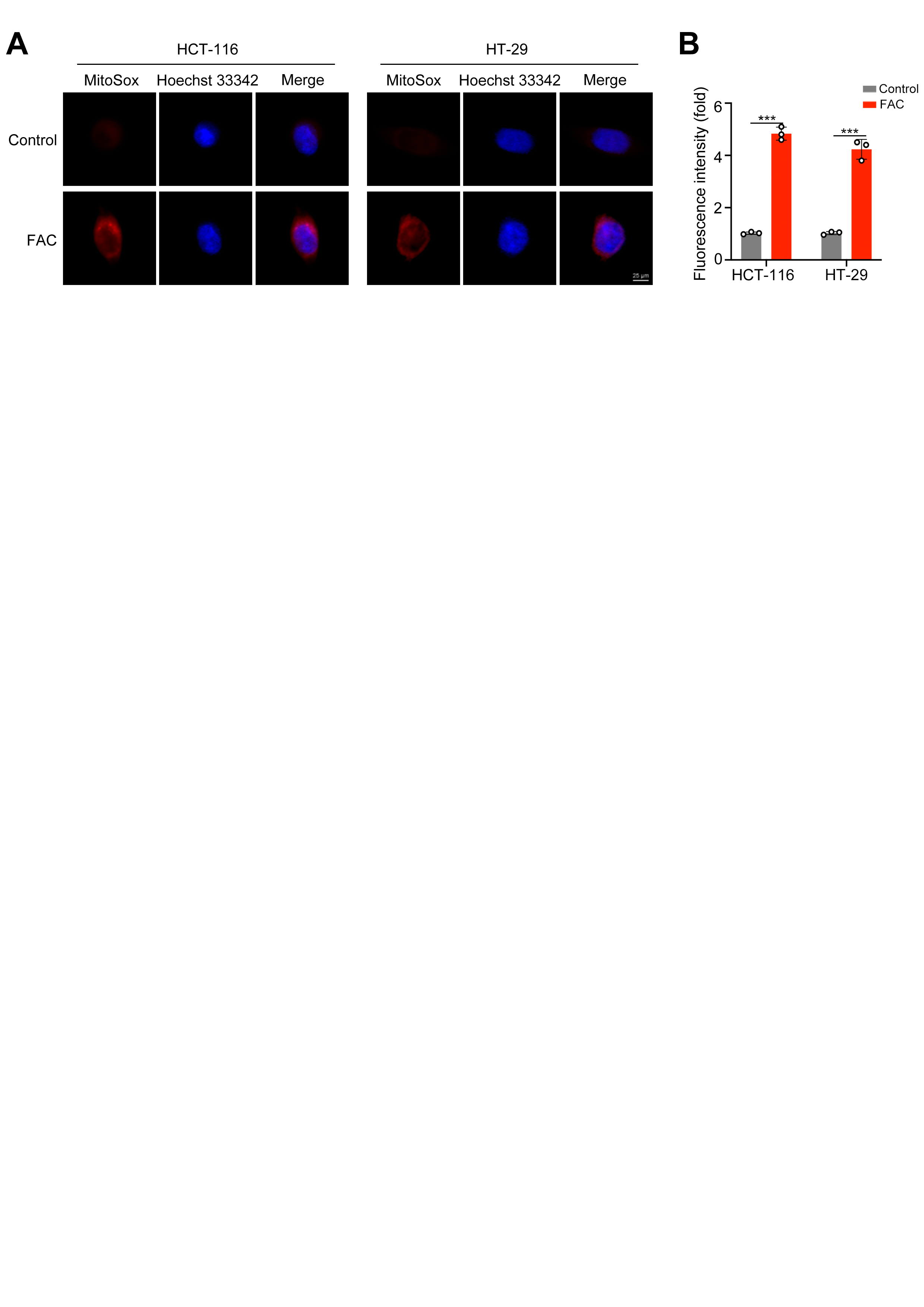

Supplement: Supplementary Figure 2 — Iron-induced ROS mainly accumulated in mitochondria. (A, B) Mitochondria ROS production detected by MitoSox (red) and Hoechst 33342 (blue) and its quantitative analysis. (***p < 0.001). [file Image_2.tif]
