# Supplementary material for: Iron Regulates the Warburg Effect and Ferroptosis in Colorectal Cancer
Source: Front Oncol. 2021 May 18;11:614778. doi: 10.3389/fonc.2021.614778 (PMC8169994; doi:10.3389/fonc.2021.614778)
Supplement: Supplementary Table 1 — Primers for qRT-PCR and sequences for cell transfection. [file Table_1.docx]

**Table S1. Primers for qRT-PCR and sequences used in cell transfection.**

| **Primers for qRT-PCR** | | | |
| --- | --- | --- | --- |
| **Gene** | **Forward Sequence (5’ – 3’)** | | **Reverse Sequence (5’ – 3’)** |
| GAPDH | TGCACCACCAACTGCTTAGC | | GGCATGGACTGTGGTCATGAG |
| LDHA | AAGCGGTTGCAATCTGGATTCAG | | GGTGAACTCCCAGCCTTTCC |
| PGK1 | CATACCTGCTGGCTGGATGG | | CCCACAGGACCATTCCACAC |
| HK2 | GAGCCACCACTCACCCTACT | | CCAGGCATTCGGCAATGTG |
| **Sequences for cell transfection** | | | |
| **Gene** | | **Sequence** | |
| shNRF2 #1 | | ATGAGTTCACTGTCAACTG | |
| shNRF2 #2 | | AGCATGCTGAAAACTTCGA | |
